# Reproductive biology of *Pittosporum* dasycaulon Miq., (Family Pittosporaceae) a rare medicinal tree endemic to Western Ghats

**DOI:** 10.1186/1999-3110-55-15

**Published:** 2014-02-02

**Authors:** Krishna Kumar Gopalakrishnan, Thuruthiyil Dennis Thomas

**Affiliations:** 1 Postgraduate and Research Department of Botany, St. Thomas College, Palai, Arunapuram (P.O.), PIN 686574 Kottayam (Dt.), Kerala India; 2 Department of Pharmaceutical Biology and Botany, Medical University in Wroclaw, Al. Jana Kochanowskiego 10, 51-601 Wroclaw, Poland

**Keywords:** Floral biology, Medicinal tree, Phenology, *Pittosporum dasycaulon*, Pollen morphology, Reproductive biology

## Abstract

**Background:**

For successful cultivation and conservation of plants a detailed knowledge of their reproductive biology is required. The reproductive features of trees are important to determine the diversity patterns and community structure of tropical forests. The present study on reproductive biology of *Pittosporum dasycaulon*, a rare medicinal tree, was conducted in the shola forests of Vaghamon hills, one of the foot hills of Southern Western Ghats of India from 2008–2011.

**Results:**

The plant flowers profusely during February to April. Inflorescence is a raceme and the total number of flowers per inflorescence varies from 96–217. The flowers are comparatively small, hermaphrodite, short pedicellate, complete, zygomorphic, pentamerous, polypetalous, hypogynous and light cream in colour with an average length of 1.14 cm. Anthesis started at 08.30 h and the flowers were completely opened at approximately 09.30 h followed by anther deshiscence at 10.00-11.30 h. The pollen grains were trizonocolpate with 45 ± 5.6 μm in size. Acetocarmine staining showed 66 ± 6% fertile pollen at the time of anther dehiscence. The number of pollen grains in an anther is 5246 ± 845 and per flower is 26230 ± 1021. The stigma is wet, non-papillate, capitate and contains a thin film of exudates under the light microscope. The superior ovary is densely covered with papillate hairs and containing 3–8 ovules. The important floral visitors include bees and butterflies. The plant is self-incompatible and an out crosser. Fruit set under open-pollination was poor (24%) with 58.3% fruits having seeds inside. There was no fruit set in manually self pollinated flowers while over 57% of the cross pollinated flowers set fruits.

**Conclusions:**

Our study presents a detailed account on reproductive biology of this medicinal tree which may help in the conservation and genetic improvement of this particular taxa.

**Electronic supplementary material:**

The online version of this article (doi:10.1186/1999-3110-55-15) contains supplementary material, which is available to authorized users.

## Background

Reproduction is the life process which ensures the perpetuation of life and genetic diversity is mainly generated through recombination processes in sexual reproduction, which is, hence, a process of fundamental importance for population and species biology (Maynard 1978). For successful cultivation and conservation of plants a detailed knowledge of their reproductive biology is required (Moza and Bhatnagar 2007). Reproductive biology mainly focuses on flowering phenology, floral biology, pollen-pollinator interaction, breeding systems and gene flow through pollen and seeds. The task of plant phenology is to observe and record the periodically recurring growth stages and to study the regularities and dependency of the yearly cycles of development on environmental conditions. In plants phenological events such as bud-burst, leaf-expansion, leaf-abscission, flowering, fertilisation, seed set, fruiting, seed dispersal and seed germination all take place in due season. Plant breeding systems extent from full self compatibility to full self incompatibility. Self incompatibility reduces the risk of inbreeding depression by limiting the number of compatible mating pairs, while self compatibility eliminates mate limitation by allowing each individual to self-fertilize. In some plants the fruit set in open pollinated flowers will be very low. This phenomenon is usually observed in hermaphroditic plants (Sutherland, 1986) which exhibit self-incompatibility. Low fruit set in nature may be largely due to a high incidence of self-pollination and a high level of selfincompatibility, but several other causes, such as resource limitation and position of fruit within inflorescences, may also be involved (Bawa and Webb, 1984).

Of the various genus of the family Pittosporaceae, *Pittosporum* alone is found in India. About 300 species of *Pittosporum* is reported out of which only eleven are observed in India. Most of the species are useful in traditional Chinese medicine for their sedative and cough relieving effects. Several active phytochemical compounds such as triterpenoid saponins, carotenoids, and essential oils were isolated from *Pittosporum* (Feng et al. 2010; Chou et al. 2008; Maoka et al. 2008; Seo et al. 2002). The antileukemia properties of *Pittosporum* had been reported recently by Cragg et al. (2006). The biological properties of *Pittosporum* has been mainly attributed to the presence of a number of volatile mono and sesquiterpenes in leaves (Chou et al. 2008).

Due to its high medicinal value, the plants are harvested in an excessive manner and thus *P. dasycaulon* needs conservation. There is only very scanty information on reproductive biology of the genus *Pittosporum*. However, some preliminary reports regarding the reproductive biology of some members of Pittosporaceae has been reported. Dioecy, self-compatibility and vegetative reproduction has been investigated in *Hymenosporum flavum* (Adam and Williams 2001). Reproductive biology, dispersal and population structure of *Pittosporum Undulatum* has been studied by Mullett (1996). An adequate understanding of the reproductive strategies of this plant requires detailed studies of their floral biology, reproductive phenology, pollination, and breeding systems. To our knowledge there is no published account on any aspects of reproductive biology or any related areas of this plant. Therefore, the present study provides a detailed account of reproductive biology i.e. phenology, floral biology, floral visitors and breeding system of *P. dasycaulon*.

## Methods

### Study species

*Pittosporum dasycaulon* (Family-Pittosporaceae) is a rare medicinal evergreen tree growing up to 12 m height and endemic to Western Ghats. Rare species have very narrow geographical distribution and highly specific habitat requirements and are restricted to small populations only (Primack 1993). *P. dasycaulon* occupied very limited in number in Western Ghats. Bark is thin, brown and the young branchlets are terete, pubescent and lenticellate. Leaves are simple, alternate, elliptic, subcoriaceous, glabrous and usually crowded at the apex. The leaf base is cuneate and the apex is acute. The population of plant is very limited in each locality and is now in the threatened category. Plant shows high medicinal properties. Stem bark and root bark are bitter and aromatic. It is an expectorant and possesses febrifuge and narcotic properties and is used to cure chronic bronchitis, leprosy, skin diseases and as an antidote for snake poisoning (Udayan et al. 2007; Yesohdharan and Sujana 2007). Recently the anti-inflammatory effect of a related species *P. tetraspermum* was confirmed in carrageenan induced oedema in albino rats (Rosakutty et al. 2010). When freshly cut, the bark emits a ginger like smell.

### Study site

The study was carried out for over three years on 4 natural populations comprising 12 plants growing in the grass land and shola forests of Vaghamon hills, Kottayam district, Kerala, India. In fact only those plants were available in the locality and no other trees of the same species were available for study. The approximate age of the marked trees varied from 10–26 years. The approximate height of the trees ranged from 5–12 meters.

### Phenology

Phenological observations were recorded monthly with respect to leaf fall (if any), leaf production, flowering (Including floral bud formation), and fruiting (Period between fruit formation until seed dispersal) on the selected plant in the studying area. The intensities of these phenological events were estimated using the semi-quantitative scale of Fournier (Fournier 1974) and identification of the morphological patterns was made according to the classification proposed by Newstrom (Newstrom et al. 1994).

### Floral biology

Flowers of *P. dasycaulon* on the day of anthesis were collected for floral biological studies. Type of inflorescence and the average number of flowers/inflorescence were registered. Length of sepals, petals, stamens and gynoecium were measured. Number of stamens, their arrangement, and time of anther dehiscence were also registered. The nature of stigma, style and the position of ovaries were identified by using a dissection microscope. Number of locules and the number of ovules per locules were registered by taking the free hand sections of ovary. Number of pollen grains per flower and number of ovules per ovary were measured by various methods given by Kearns and Inouye (Kearns and Inouye 1993). Pollen size was measured with an ocular micrometer under light microscope following the procedure of McKone and Webb (McKone and Webb 1988). Pollen morphology was determined by acetolysis (Shivanna and Rangaswamy 1993). In acetolysis the pollen grains were treated with acetic anhydride and sulfuric acid to dissolve the cellulistic materials present on the surface of the pollen grains and provide better visibility for studying pollen sculpering. Photographs were taken by using a photomicroscope (Labomed, India) attached to a camera (Nikon D70).

### Pollen/ovule (P/O) ratio

Pollen ovule (P/O) ratio was verified in pre-anthesis buds by dividing the number of pollen grains by the number of ovules/flower (Cruden 1977). The number of ovules in the ovaries were counted with the help of a dissecting microscope (Nikon). Buds examined for P/O ratio were near anthesis i.e. pollen was mature but the anther has not dehisced. The number of buds collected from each specimen varied from 12–20.

### Reproductive biology

Pollen viability tests were conducted by using pollen grains from 25 newly opened bagged flowers. *In vitro* hanging drop culture method (Brewbaker and Kwack 1963) was used to check the pollen viability. The composition of the Brewbaker and Kwack’s medium includes boric acid (100 mg/l), calcium nitrate (300 mg/l), Magnesium sulfate (200 mg/l), potassium nitrate (100 mg/l) and sucrose (10%). The hanging drop method consisted of suspending the pollen grains in a drop of nutrient medium on a coverglass hanging over a shallow depression. The hanging drop culture was sealed with petroleum jelley to prevent evaporation of the culture medium. Time taken for pollen tube germination, and the average length of pollen tubes were measured. Pollen load on stigmatic surface were checked by treating the pistil in 8 N NaOH. Presence or absence of self incompatibility was also verified by pollen load on stigmatic surface and pollen tube germination on stigmatic surface. Fruit set and seed set were observed every week after pollination.

### Breeding systems

The breeding system of *P. dasycaulon* was conducted by controlled pollination done in flowers that were previously enclosed with paper bags still in the bud pre-anthesis phase to avoid any kind of natural pollination. Various types of experiments including self-pollination and cross pollinations were carried out (Radford et al. 1974). For controlled pollination, emasculation was carried out as follows. Flower buds were selected about 1–2 days before anthesis, opened carefully and excised all anthers with a forceps, bagged and carefully labelled. On the day of anthesis, the controlled pollinations were carried out. For self-pollination experiments bagged flowers without emasculation were manually pollinated with the same pollen. For cross pollination, emasculated flowers were pollinated with pollen from various other plants. For natural pollination experiments, undisturbed naturally growing flowers were used. The bags were removed from the flowers only after the anthesis and the fruit development was monitored every week. In addition to above treatments, the flowers were also marked to evaluate the percentage of fruit set under natural conditions. We also calculated the reproductive efficacy (the ratio between the percentage of fruits formed by natural pollination and the percentage of fruits formed by hand cross pollination; Bullock 1985). In order to observe pollen tube growth, flowers were hand cross pollinated and the pistils were fixed in FAA (Formaline : acetic acid : alcohol; 5:5:90) at intervals of 8, 16, 24, 48, and 72 h after pollinations, studied for pollen tube growth.

### Floral visitors

During the flowering period detailed studies on floral visitors were done. All the plants were observed in the field for more than 20 hours each during the field trips. Floral visitors and their behaviour were recorded from 09:30 AM to 15:00 PM. Type of floral visitors, purpose of visiting, interaction with flowers (which includes attempt to visit flower i.e. flies close without touching the flower, direct contact and effect pollination) were properly recorded. Some of the visitors were captured for identification.

## Results

### Phenology

**Figure 1:**
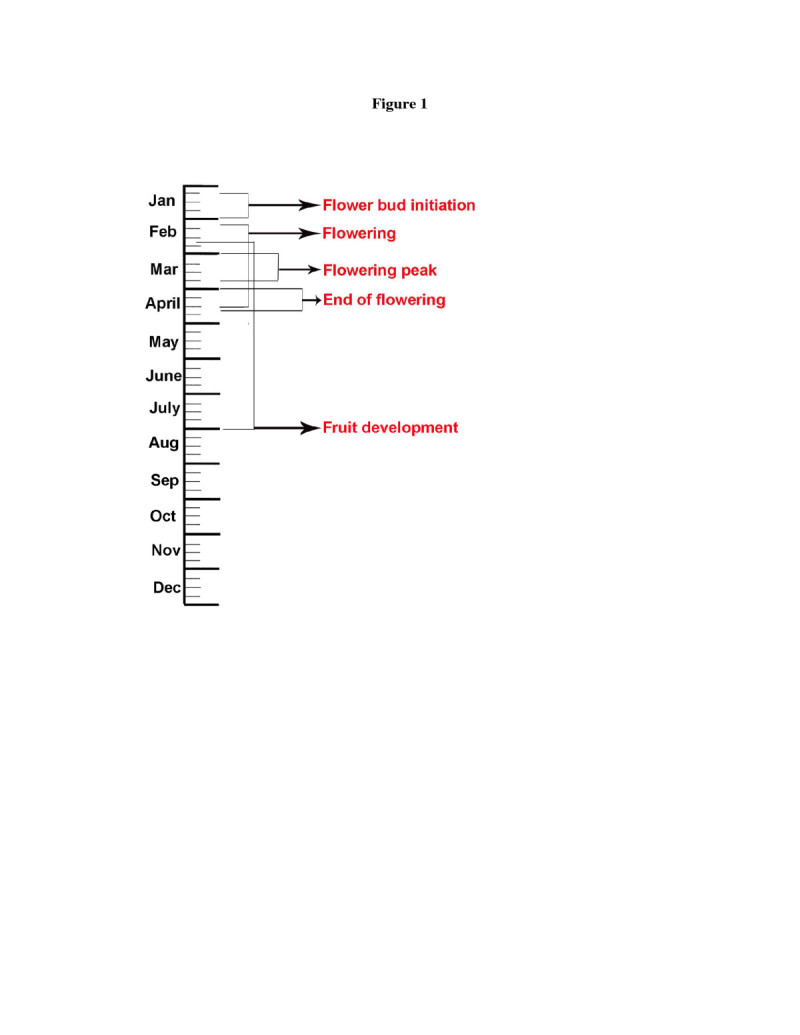
**Different phenological events in** ***P. dasycaulon*** **.** The trees are clothed with the foliage throughout the year since the plant is an evergreen tree. Flowering lasts about three months and fruiting period extends over five months.

**Figure 2:**
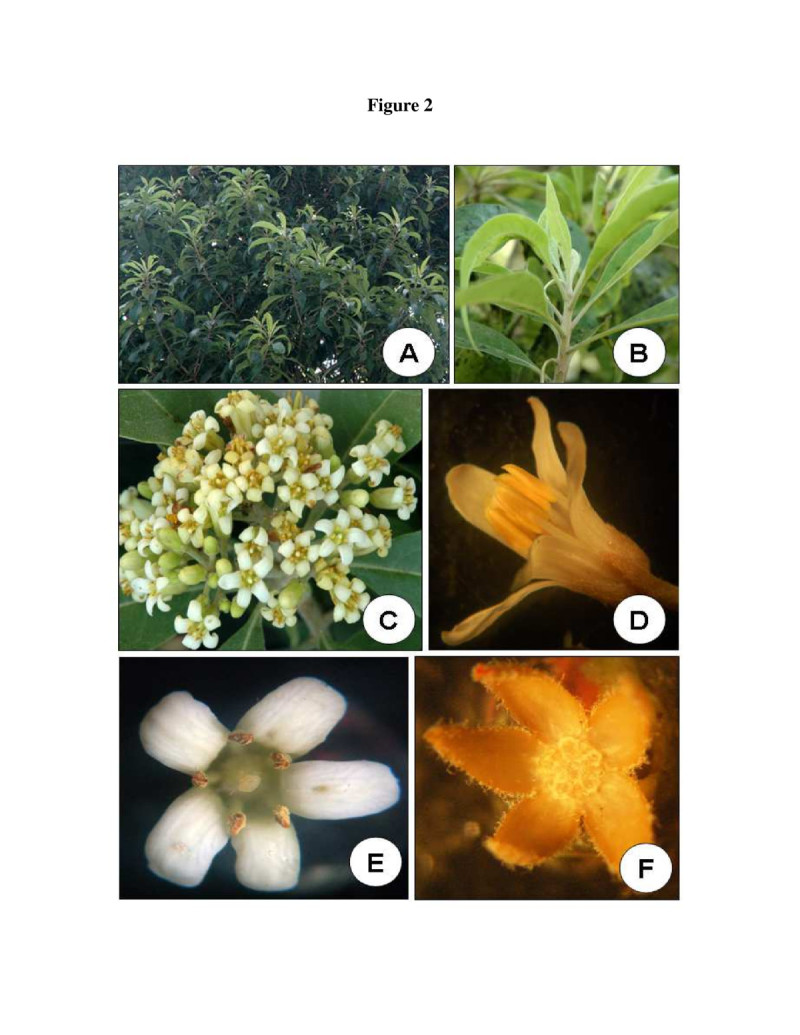
**The plant and flower of** ***P. dasycaulon*** **. A**. A tree growing in the study site during the growth period. **B**. New flush developing before the onset of flowering from the apical regions of the plant. The colour of newly emerged leaf is light green. **C**. An inflorescence during flowring peak. **D**. A single flower during anthesis. The anthers are not dehisced. **E**. A single flower 12 hour after anthesis. The anthers have dehisced. **F**. Calyx after removing all other floral parts.

Figure 1 presents the details of phenological events. In *P. dasycaulon* total loss of foliage was never observed as the plant is an evergreen tree. The plant was covered fully with green leaf throughout the year (Figure 2A). The average leaf amount was uniform throughout and there was no leaf shedding season. About one month before flowering new leaves sprouted from the tip of the buds. These new leaves appeared light green (Figure 2B) as compared to mature leaves which remained dark green. The flower buds started the development during the second week of January. Although the flowering started in February in most of the populations, some intermittent flowering occurred during January in some populations. But these flowers did not produce any fruits. Flowering started in February and continued till the third week of April (Figure 2C). Flowering peak was observed from the first week of March to fourth week of March. Flowering showed a decline from the first week of April and complete disappearance of flowers were found in the third week of April. In some plants the flowering continued up to the month of July. However, these flowers produced very less fruits (less than 2%). Fruit development started early in the first formed flowers. Maximum flower to fruit ratio was observed during the peak flowering period (19%). Fruit development took more time and average time required for flowering to fruit dehiscence took about 3 months. Some fruits were observed on the plant even during the month of August. Since the fruits were dehiscent, the mature fruits were dehisced and the seeds were dispersed automatically.

### Floral biology

**Figure 3:**
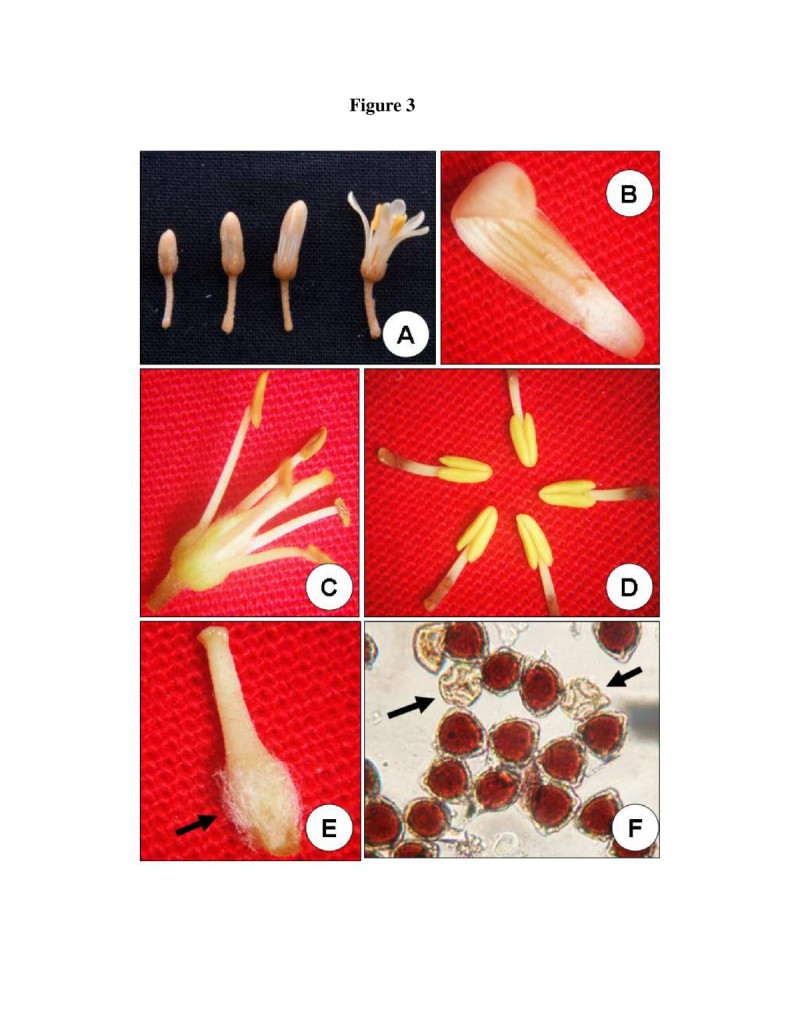
**Characteristic of flower in** ***P. dasycaulon*** **. A**. Flower bud at various stages of development. The last figure shows a flower at the time of anthesis. **B**. A single petal excised from the flower after anthesis. **C**. Figure showing stamens and pistil 4 hour after anthesis after removing all other floral parts. **D**. Anthers removed from a single flower at anthesis. **E**. An excised pistil showing stigma, style and ovary. Note luxurently growing papillate hairs at the basal region of the ovary. **F**. Pollen sterility assessed by acetocarmine staining. Arrows indicate the sterile pollens.

The floral traits of *P. dasycaulon* are presented in Table 1. Inflorescence is a terminal raceme (Figure 2C) and the total number of flowers per inflorescence ranged from 96–217. Each day an average number of 10 ± 2 flowers per inflorescence opened at the peak of the flowering period. Inflorescence had several small branches and the flowers were arranged in an acropetal succession on it. Flowers were small, short pedicellate, complete, zygomorphic, bisexual, pentamerous, polypetalous, hypogynous and light cream in colour with an average length of 1.14 cm (Figure 2D and E). The flower had a pungent odour. Calyx consisted of five free sepals with an average length of 0.19 cm (Figure 2F). Corolla consisted of five whitish cream coloured petals with an average length of 0.78 cm (Figure 3B). It took about three weeks from flower bud initiation to flower anthesis (Figure 3A). Anthesis started at 08:30 AM and the flowers were completely opened by approximately 09:30 AM.

**Table 1:** **An overview of various floral traits such as type of inflorescence, flowering period, type of flower, colour of flower, odour of flower, presence of nector, flower opening time, anther dehiscence time, number of anthers per flower, average number of pollen grains per flower, average number of ovules per ovary, pollen-ovule ratio, type, shape, size and viability of pollen and stigma type of** ***P. dasycaulon***

Parameters	Observations
Inflorescence	Simple raceme
Flowering period	February-April
Flower	Hermaphrodite and zygomorphic
Flower colour	Cream
Odour	Mild fragarance
Nectar	Nil
Flower opening	8:30–9:30 AM
Anther dehiscence	Bursting inwards by slits
Number of anthers/flower	5
Average number of pollengrains/flower	20800
Average number of ovules/ovary	3-8
Pollen-ovule ratio	2600:01:00
Pollen type	Trizonocolpate
Pollen shape	Triangular
Pollen size	45 ± 5.6 μm
Pollen viability	64 ± 4%
Stigma type	Capitate

The androecium consisted of five stamens present in the form of a ring around the carpel and they were present at the same level of stigma (Figure 3C). Average length of stamen was 0.72 cm (filament = 0.23 cm, and anther = 0.49 cm; Table 2). Anthers were basifixed and yellowish while the filament was short and creamy in colour (Figure 3D). The yellowish anthers dehisced between 10:00–11:30 AM. Anther dehiscence took place through a longitudinal slit. Usually the anthers dehisced approximately 30 minutes after flower anthesis and shed 90% of the pollen within 2–3 hours. The flowers that do not set fruits abscised within 3–4 days. The petals wilted and fell down and the stamens dried from the basal part of the ovary. The ovary remained attached to the pedicel after pollination but the apical portion of the pistle i.e. stigmatic portion, dried up.

**Table 2:** **Flower characteristics in** ***P. dasycaulon***

Parameters studied	Measurement*
Flower length	1.14 ±0.04 cm
Length of calyx	0.19 ± 0.03 cm
Length of corolla	0.78 ±0.05 cm
Length of stamen	0.72 ± 0.04 cm
Length of pistil	0.69 ±0.05 cm

*Average of 8 flowers from each plant.

**Figure 4:**
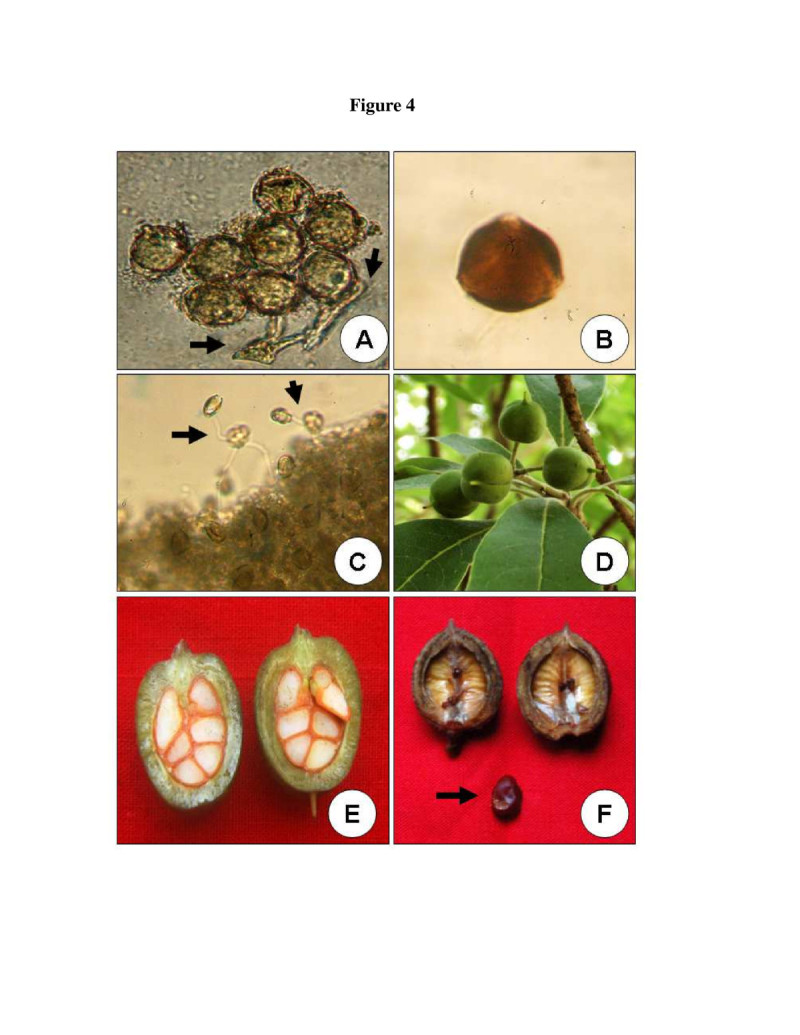
**Pollen germination and fruit in** ***P. dasycaulon*** **. A**. In vitro pollen germination. Arrows indicate the germinating pollen and pollen tube. **B**. A single pollen after acetolysis. Note the trizonocolpate nature of the pollen. **C**. Pollen germination on stigmatic surface observed under normal microscope after hydrolysing the pistil. **D**. Four mature fruits growing on a tree from study site. **E**. Longitudinal section of a single fruit showing 6 red seeds inside. **F**. A dehisced fruit with a single seed.

Acetolysis of the pollen revealed the pollen morphology. The pollen grains were triangular and trizonocolpate with 45 ± 5.6 μm in size (Figure 4B). Pistil was white cream in colour and differentiated into stigma, style and ovary, having an average length of 0.78 ± 0.4 cm at anthesis (stigma = 0.026 ± 0.003 cm, style = 0.35 ± 0.02 cm, ovary = 0.41 ± 0.04 cm; Figure 3E). The stigma was wet, non-papillate and capitate. Stigma contained a thin film of exudates under the light microscope at the time of anthesis. Style presented medium size with average length of 0.35 ± 0.02 cm. Superior ovary was densely covered with papillate hairs and contained 3–8 ovules (Figure 3E).

### Pollen/ovule (P/O) ratio

The pollen:ovule ratio (P/O) has traditionally been widely used as a rough estimator of breeding system studies. It has been shown that plant breeding systems are associated with particular floral traits. Pollen and ovule numbers per flower estimated in 2008 to 2011 were used to calculate the pollen:ovule ratio. Total pollen production per flower averaged 20800 and the number of ovules per flower varied between 3 to 8. In the present investigation the pollen ovule ratio of *P. dasycaulon* was 2600:1 (Table 1).

### Reproductive biology

**Figure 5:**
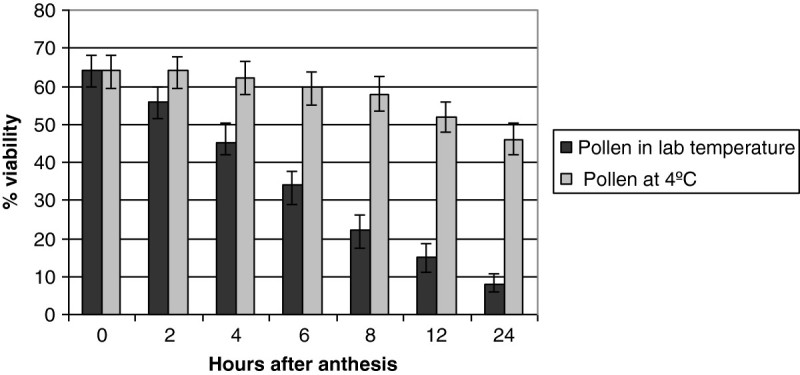
**Viability of pollen stored at lab temperature and at 4°C in relation to time after flower anthesis (indicated as 0).**

Acetocarmine staining showed 66 ± 6% fertile pollen at the time of anther dehiscence (Figure 3F). Studies with pollen germination in vitro revealed that the pollen grains viability was 64 ± 4% at the time of anthesis (Table 1; Figure 4A). Pollen viability showed a steady decrease 4 h after storage in the laboratory (25 ± 2°C) conditions and viability reached less than 15 ± 2% 12 h after storage in lab (25 ± 2°C) conditions. However, 46 ± 3% of the pollen grains stored at 4°C in a refrigerator remained viable after 24 h (Figure 5). The number of pollen grains in an anther was 5246 ± 845 (mean ± SD) and per flower was 26230 ± 1021 (mean ± SD).

### Fruit set

Details of fruit set by various pollination experiments are presented in Table 3. Fruit set under open-pollination was poor and only 24% of the flowers set fruits with 58.3% fruits having seeds inside. None of the emasculated and bagged flowers as well as those bagged without emasculation sets fruits (Table 3). Therefore, it is confirmed that in this species there is no apomixy or autogamy. Additionally, none of the manually self pollinated flowers formed fruits, while over 57% of the cross pollinated flowers formed fruits.

**Table 3:** **Fruit and seed set in treated flowers**

Treatment	No of flowers pollinated	No. of fruits developed (%)	No of fruits having seeds (%)
Bagged without emasculation	35	0.0	-
Bagged after emasculation	35	0.0	-
Self-pollination	35	0.0	-
Cross pollination	35	20 ± 2.3 (57)	16 ± 2.1 (80)
Natural pollination	50	6.0 ±1.8 (12)	4 ± 0.6 (66.6)

Values indicate the average of three independent experiments done in 12 plants.

The average flower and fruit production during the study period of 12 labelled individuals were presented in Table 4. The average number of total flowers per plant varies in each year. Average number of total flowers per plant were 18102, 15438 and 23541 and ripening fruits per plant were 2302, 2234 and 2613 during the year 2009, 2010 and 2011 respectively (Table 4). However, the number of mature fruits per plant was 908, 838 and 1203 during the year 2009, 2010 and 2011 respectively. Fruit was a globose, glabrous capsule with 1.0 to 1.5 cm in diameter (Figure 4D). Mature seeds were dark brown in colour and covered with a resinous viscous fluid (Figure 4E). The ovoid capsule dehisced by 2 equal valves (Figure 4F). Number of seeds varied from 3–8. However, most of the seeds contained 3–4 seeds only. Seeds measured about 5.0 mm in diameter (Figure 4F).

**Table 4:** **Average flower and fruit production per plant during the study period of 12 labelled individuals**

Year	Average number of flowers/plant	Average number of ripening fruits/plant	Average number of mature fruits/plant
2009	18,102 ± 1041	2302 ± 123	908 ± 32
2010	15, 438 ± 1062	2234 ± 76	838 ± 32
2011	23,541 ± 1232	2613 ± 112	1203 ± 21

### Floral visitors

The flowers were visited by honey bees, *Apis dorsata* and *Apis indica* and butterflies. Honey bees and butterflies visited the flower in the morning after anthesis. All these visitors played significant role in cross-pollination.

**Figure 6:**
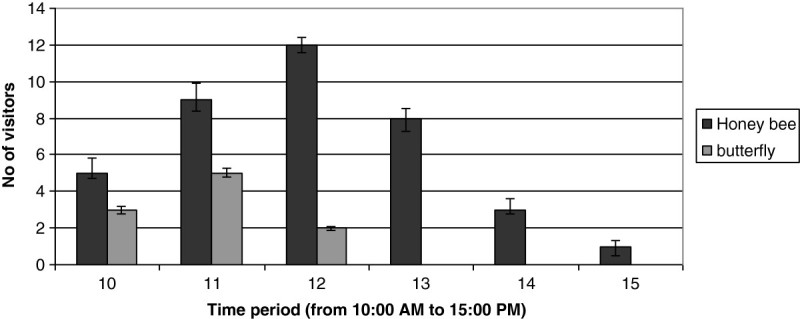
**Average number of floral visitors in 12 different plants studied during the peak flowering period.**

An average number of floral visitors in 12 different plants studied during the peak flowering period were presented in Figure 6. The maximum floral visitors (12 honey bees and 2 butterflies) were recorded during 11:00–12:00 AM. Based on our observations it can be realized that the effective pollinators are honey bees and butterflies. Cream colour and odour of flowers attracted the pollinators towards the flower after anthesis. Occasionally it was noticed that ants, house flies and spiders also visited the flowers during afternoon between 2:00 PM-4:00 PM. However, on the basis of visitation rates, transfer of pollen on virgin stigma and pollen load on body parts of insects, it was confirmed that honey bees and butterflies are the effective pollinators in this plant.

## Discussion

The basic knowledge on reproductive biology is not only essential for evolutionary and systematic studies (Anderson 1995) but also important for effective conservation strategies (Holsinger 1991; Bernardello et al. 1999) for endemic and threatened plants with very few populations like *P. dasycaulon.* The most crucial stages in the life cycle of any plant include reproduction, dispersal of seeds, germination followed by seedling establishment (Kavanagh and Carleton 1990). Reaching the reproductive phase is an important step in the life cycle of a plant as far as resource allocation is concerned since the assimilates reserved previously for vegetative growth will be utilized for the generative purposes (Seifert and Muller-Starck 2009).

From our study we found that *P. dasycaulon* has an annual flowering pattern with a single major flowering episode which has been reported in some other species like *Caesalpinia echinata* (Borges et al. 2009). Fresh leaf flesh emergence prior to flowering observed in our system is a typical pattern observed in several tree species growing in various habitats (Bullock 1995). During the flowering to fruiting period there was slight asynchrony among the studied plants. Such asynchrony among individuals may be due to microhabitat differences as reported by Newstrom et al. (1994). Detailed information on phenological studies helps in the conservation as well as framing effective measures for successful cultivation of a species (Delanoe et al. 1996; Wafai et al. 1996).

*P. dasycaulon* is a self incompatible plant with comparatively low reproductive output. The predominant self-incompatibility nature of forest trees is directly related to selective pressure to maintain genetic variability (Bawa 1974). Variations especially genetic variation has long term evolutionary consequence and form the basis of the raw material for natural selection to operate and exploitation in the selection of superior genotypes at the hands of a breeder. Self-incompatibility is the most efficient method of controlling self pollination and thereby inbreeding in higher plants (Mandujano et al. 2010). Hand pollination resulted in more fruit set than natural pollination and this confirm that there is pollination deficit in this species and is mainly attributed to the lack of pollinators or lower effectiveness of the pollinators. In this species the main pollinators are honey bees and are known for less effective pollinators as compared to other bee species since they are more generalist and they collect pollen from various pollen resources resulting in the heterospecific pollen on the stigmatic surface (Westerkamp 1991). Habitat loss and excessive pesticide usage may be the reasons for low number of pollinators (Kearns and Inouye 1997). Forest destruction and fragmentation may often cause damage to diverse pollinators. Highly mobile species may be more resistant to habitat fragmentation than other species (Steffan and Tscharntke 1999; Tilman 1994).

Pollen viability test, percentage of pollen germination and pollen load on stigmatic surface gave a clear cut evidence that *P. dasycaulon* is a self-incompatible species under natural condition. Although the number of pollen grains are more, only very few are viable in *P. dasycaulon*. But viable pollen grains had done effective fertilization. Low temperature often influenced the pollen tube germination.

Pollen production in a plant depends upon several factors including season, anther length, pollen grain size and mode of anther dehiscence (Stanley and Linskens 1974). Recent studies showed that the effects of pollen quantity and quality on reproductive success have become an important issue in plant conservation (Dudash and Fenster 2000; Rocha and Aguilar 2001; Byers 2004; Colling et al. 2004) as they are closely related to demographic and genetic factors that affect population decline and extinction. In the present study, in vitro pollen germination studies showed moderate pollen viability (64%). In vitro studies often provide lot of information on physiological and biochemical nature of pollen germination and pollen tube growth on stigmatic surface (Shivanna and Johri 1985).

The fruit set for all plants studied were low with low fruit to flower ratios. This may be due to changes in various climatic conditions including rainfall, temperature variations and humidity resulting in altered physiological condition of the plant. The failure of high rate of transformation from flower to fruit indicates that the fruit set in this plant is resource limited (Corbet 1998; Fleming and Holland 1998). The probable other reasons for reduced fruit set include reduced pollen viability, low pollen loads on stigmas, the absence of pollen tubes at the base of the style, small population size, space limitation, genetic load and climatic change which can cause low fruit set (Lamont et al. 1993; Hermanutz et al. 1998; Dorken and Eckert 2001). However, specific studies were not conducted to examine which factor/s are more important in determining low fruit set in *P. dasycaulon*. Low fruit production might indicate low efficiency of the pollination syndrome (Whelan and Goldingay 1989).

*P. dasycaulon* is a stable self-incompatible plant. This is evident from the pollination experiments as 57% of the cross pollinated flowers set fruits and seeds.

The low fruit set in natural pollinated flowers as compared to artificial cross pollinated flowers strongly suggest the requirement of some external agents necessary for effective pollination (Sreekala et al. 2008). Others are of the view that the low fruit:inflorescence ratio is related to the physiological incapacity of the mother plant to generate and ripe as many fruits as flowers (Koptur 1984; Ruiz and Arroyo 1978). Moreover the perennial trees must interact with the environmental conditions at all times of the year, and flowering and fruiting is closely related to seasonal climatic changes (Sedgley and Griffin 1989).

Pollination success basically relies on the availability of pollen and pollinator. All the *P. dasycaulon* plants investigated in this study exhibited high levels of pollinator dependence. The cream colored corolla and the floral scent attract the insects especially honey bees. It is reported that insects especially honey bees locate flowers by visual clues and to orient during landing by floral fragrance cues (Dobson et al. 1999; Keven 1983). Honey bees, and butterflies were observed during the entire flowering period and were effective pollinator as they collected pollen and transferred on the stigmatic surface.

## Conclusions

A detailed account of various aspects of reproductive biology such as phenology, floral biology, pollen/ovule (P/O) ratio, breeding systems, floral visitors were investigated in the present study. The Flowers in *P. dasycaulon* is comparatively small, hermaphrodite, short pedicellate, complete, zygomorphic, pentamerous, polypetalous, hypogynous and light cream in colour. The flower anthesis started at 08.30 h and anther deshiscence at 10.00-11.30 h. The stigma is wet, non-papillate, capitate and contains a thin film of exudates. Our study revealed the presence of some floral visitors like bees and butterflies. From our study it is confirmed that the plant is self-incompatible and an out crosser. This detailed investigation on reproductive biology is important because, the evolutionary success and survival of this plant is largely determined by the efficacy of their reproductive performance.

## Authors’ information

TDT is the Assistant Professor and KKG is the project fellow in Department of Botany, St. Thomas College, Palai, Kottayam, Kerala, India.

## Authors’ original submitted files for images

Below are the links to the authors’ original submitted files for images. Authors’ original file for figure 1 Authors’ original file for figure 2 Authors’ original file for figure 3 Authors’ original file for figure 4 Authors’ original file for figure 5 Authors’ original file for figure 6
