# Supplementary figures and images for: Reproductive biology of Pittosporum dasycaulon Miq., (Family Pittosporaceae) a rare medicinal tree endemic to Western Ghats
Source: Bot Stud. 2014 Feb 2;55:15. doi: 10.1186/1999-3110-55-15 (PMC5432743; doi:10.1186/1999-3110-55-15)

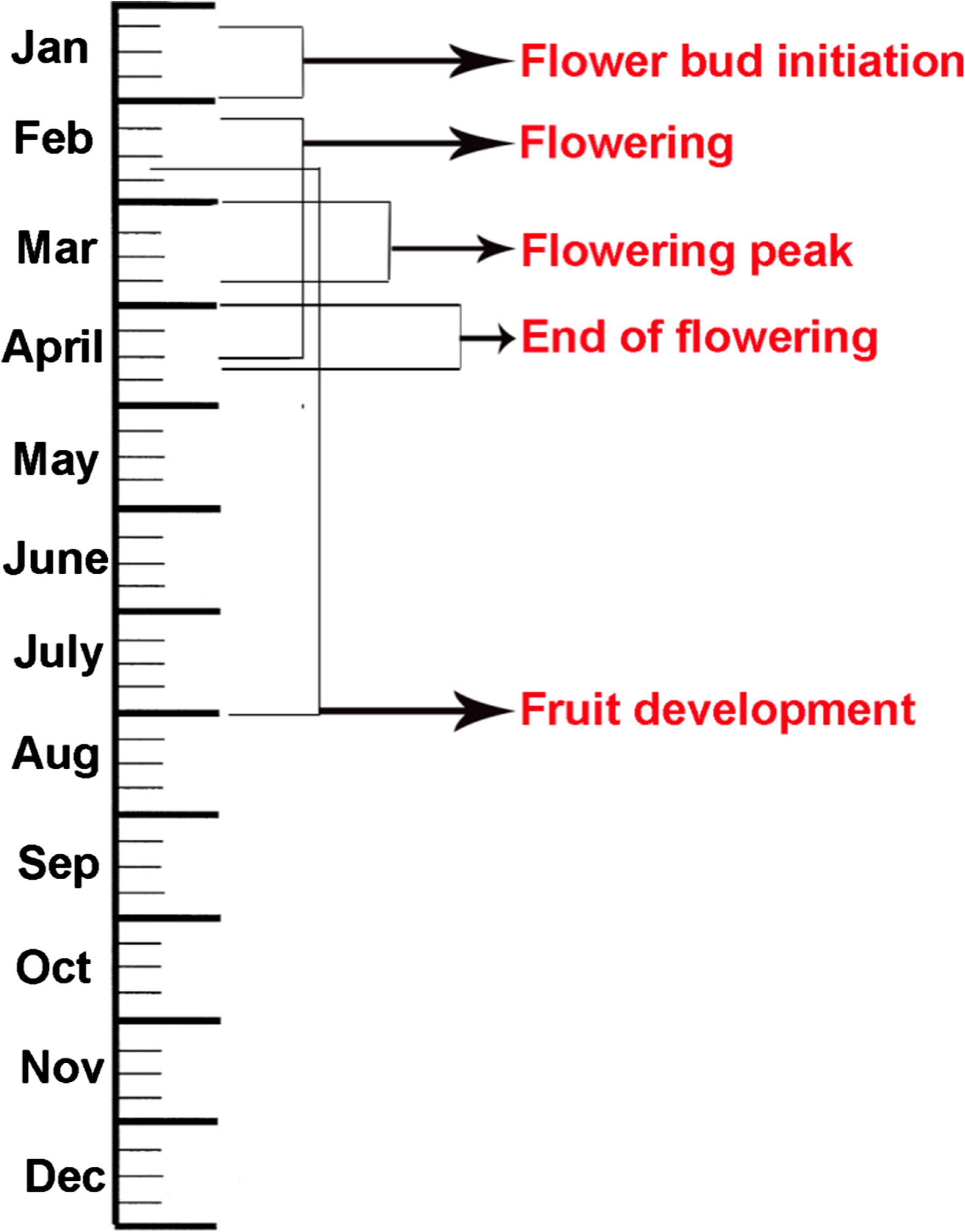

Supplement: Supplementary file 1 — Authors’ original file for figure 1 [file 40529_2013_64_MOESM1_ESM.tif]

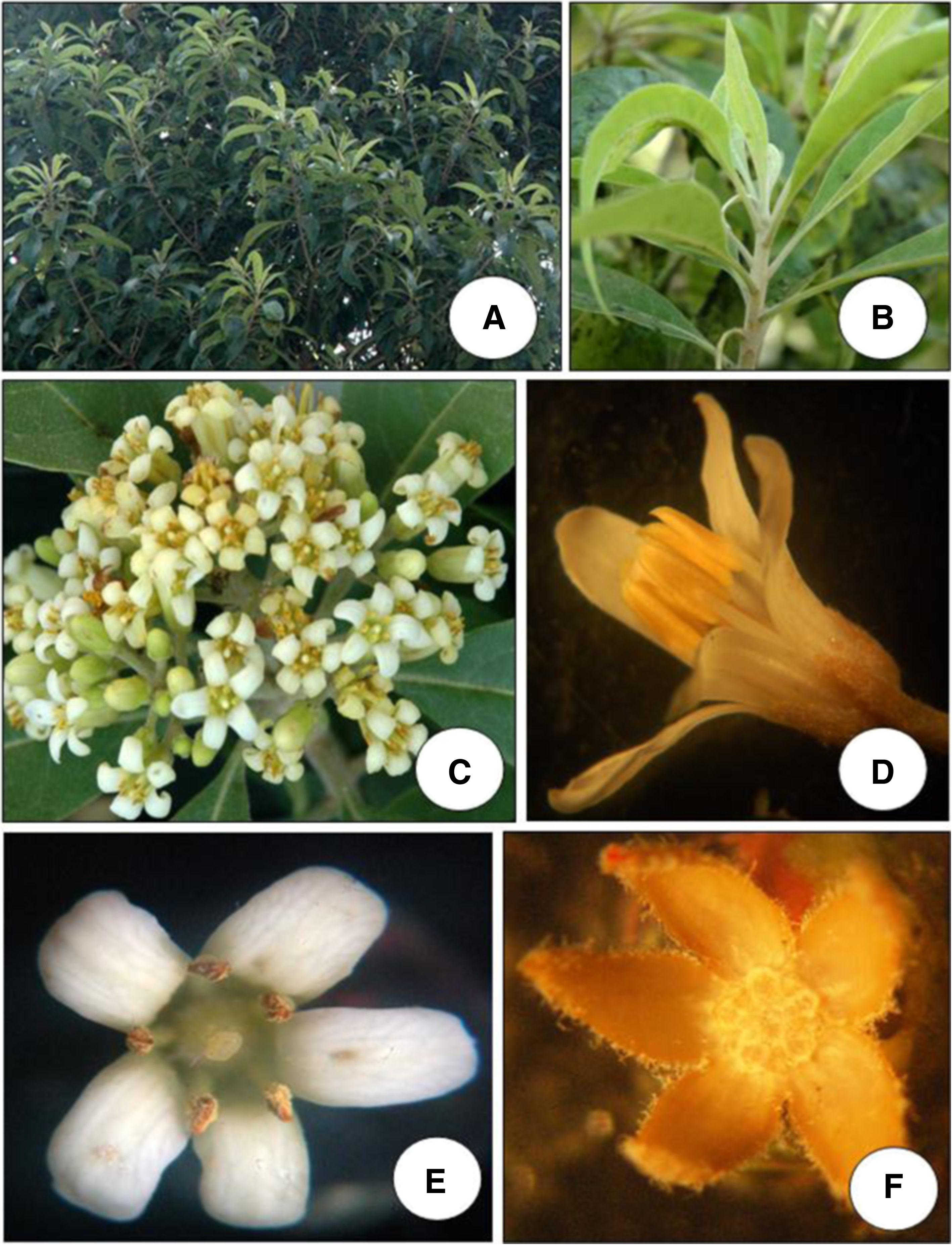

Supplement: Supplementary file 2 — Authors’ original file for figure 2 [file 40529_2013_64_MOESM2_ESM.tif]

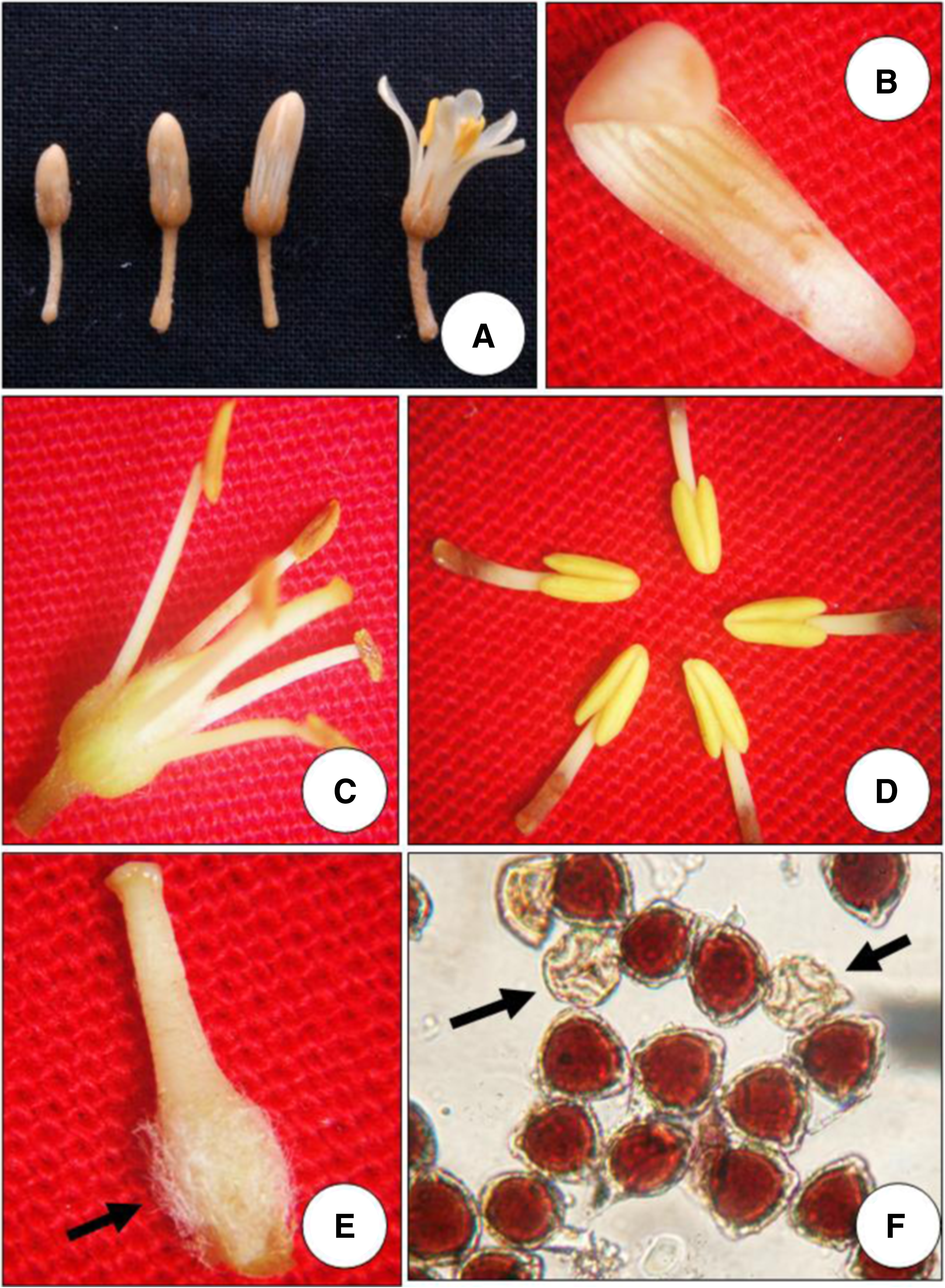

Supplement: Supplementary file 3 — Authors’ original file for figure 3 [file 40529_2013_64_MOESM3_ESM.tif]

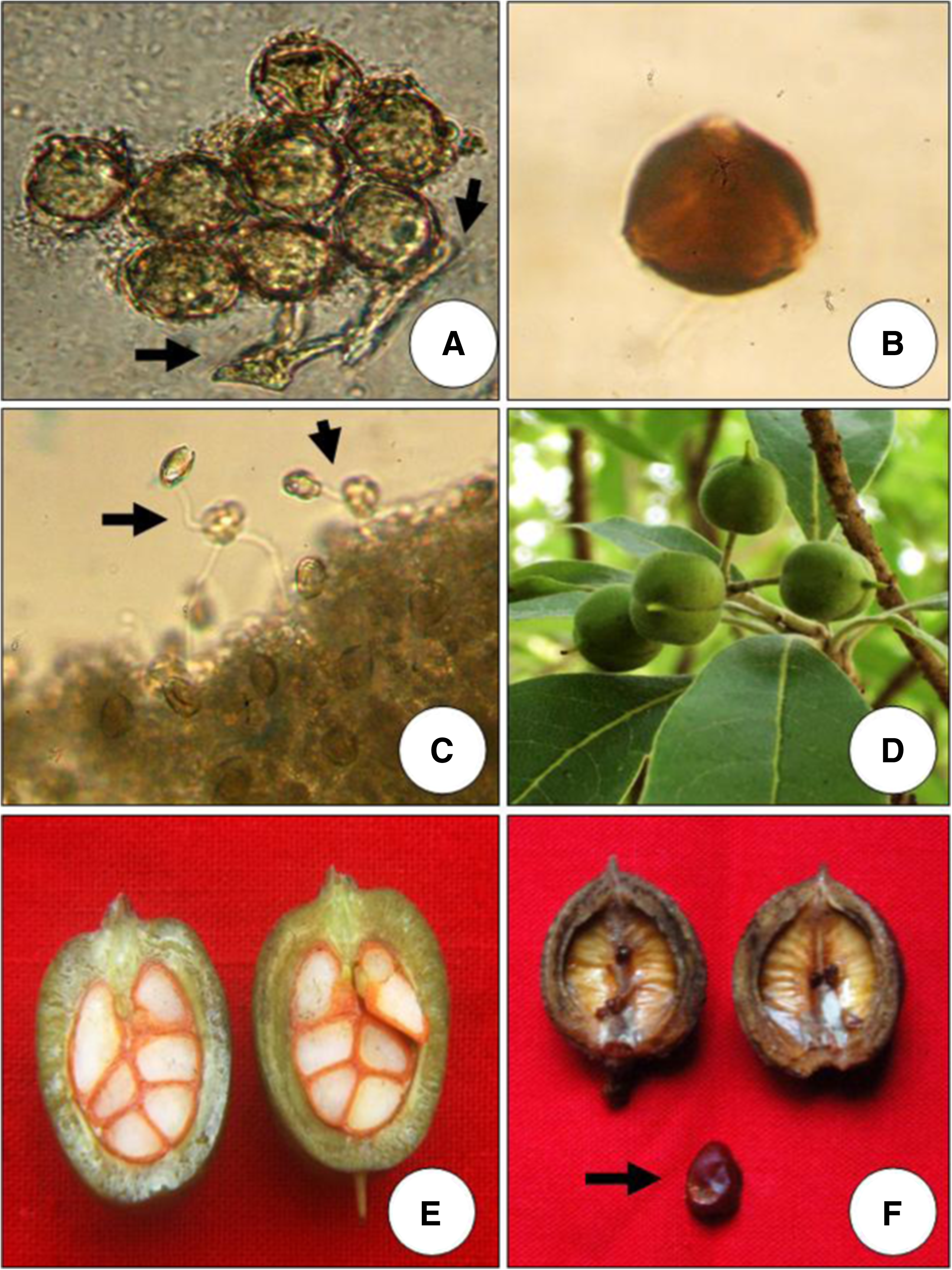

Supplement: Supplementary file 4 — Authors’ original file for figure 4 [file 40529_2013_64_MOESM4_ESM.tif]

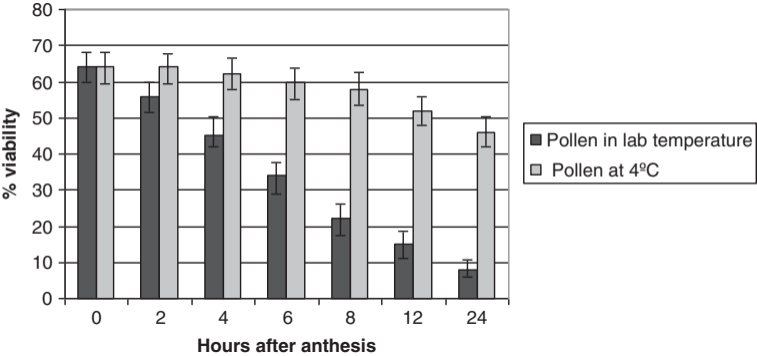

Supplement: Supplementary file 5 — Authors’ original file for figure 5 [file 40529_2013_64_MOESM5_ESM.pdf]

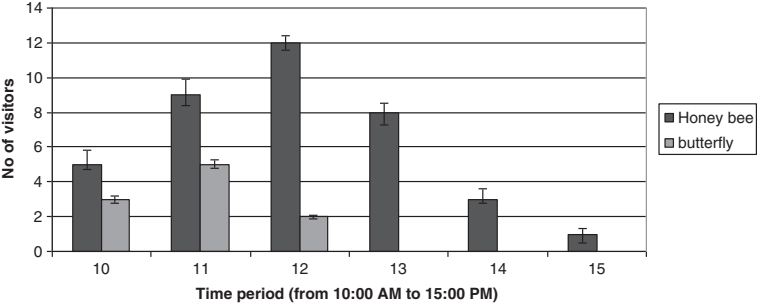

Supplement: Supplementary file 6 — Authors’ original file for figure 6 [file 40529_2013_64_MOESM6_ESM.pdf]
